# Providing safe care for patients in the coronavirus disease 2019 (COVID-19) era: A case series evaluating risk for hospital-associated COVID-19

**DOI:** 10.1017/ice.2021.38

**Published:** 2021-04-05

**Authors:** Elizabeth B. Habermann, Aaron J. Tande, Benjamin D. Pollock, Matthew R. Neville, Henry H. Ting, Priya Sampathkumar

**Affiliations:** 1 Division of Health Care Delivery Research, Mayo Clinic, Rochester, Minnesota; 2 Division of Infectious Diseases, Mayo Clinic, Rochester, Minnesota; 3 Department of Quality, Experience, and Affordability, Mayo Clinic, Rochester, Minnesota; 4 Department of Cardiovascular Medicine, Mayo Clinic, Jacksonville, Florida

## Abstract

**Objective:**

We evaluated the risk of patients contracting coronavirus disease 2019 (COVID-19) during their hospital stay to inform the safety of hospitalization for a non–COVID-19 indication during this pandemic.

**Methods:**

A case series of adult patients hospitalized for 2 or more nights from May 15 to June 15, 2020 at large tertiary-care hospital in the midwestern United States was reviewed. All patients were screened at admission with the severe acute respiratory coronavirus virus 2 (SARS-CoV-2) polymerase chain reaction (PCR) test. Selected adult patients were also tested by IgG serology. After dismissal, patients with negative serology and PCR at admission were asked to undergo repeat serologic testing at 14–21 days after discharge. The primary outcome was healthcare-associated COVID-19 defined as a new positive SARS-CoV-2 PCR test on or after day 4 of hospital stay or within 7 days of hospital dismissal, or seroconversion in patients previously established as seronegative.

**Results:**

Of the 2,068 eligible adult patients, 1,778 (86.0%) completed admission PCR testing, while 1,339 (64.7%) also completed admission serology testing. Of the 1,310 (97.8%) who were both PCR and seronegative, 445 (34.0%) repeated postdischarge serology testing. No healthcare-associated COVID-19 cases were detected during the study period. Of 1,310 eligible PCR and seronegative adults, no patients tested PCR positive during hospital admission (95% confidence interval [CI], 0.0%–0.3%). Of the 445 (34.0%) who completed postdischarge serology testing, no patients seroconverted (0.0%; 95% CI, 0.0%–0.9%).

**Conclusion:**

We found low likelihood of hospital-associated COVID-19 with strict adherence to universal masking, physical distancing, and hand hygiene along with limited visitors and screening of admissions with PCR.

The coronavirus disease-2019 (COVID-19) pandemic has drastically affected the provision of health care in the United States beginning in February 2020. Hospitals cancelled elective surgeries and procedures to prepare for this unprecedented pandemic and to conserve intensive care unit (ICU) beds, ventilators, personal protective equipment (PPE) and other resources.^1–3^ Patients decided to delay medical care,^4^ including emergency visits,^5,6^ due to concerns about severe acute respiratory coronavirus virus 2 (SARS-CoV-2) transmission in the hospital setting.

As COVID-19 rates decreased in certain regions and resources including PPE, ICU beds, and ventilators were better managed, some hospitals resumed elective surgeries and procedures for patients with non–COVID-19 indications. Furthermore, hospital infection control policies such as universal masking of hospital employees, patients, and limited visitors decreased the risk of healthcare workers (HCWs) contracting COVID-19. Previous reports have described nosocomial COVID-19 occurring in hospitals,^7,8^ but the incidence rate and risk of patients contracting COVID-19 in our hospitals were not determined.

In this study, we evaluated the risk of patients contracting COVID-19 during their hospital stay for a non–COVID-19 indication. The results of this surveillance study for healthcare-associated COVID-19 in our patients will contribute to the safety of hospitalizations during this COVID-19 pandemic.

## Methods

### Mayo Clinic Nosocomial COVID-19 Surveillance Task Force

In late April 2020, Mayo Clinic formed a Nosocomial Surveillance Task Force was formed, composed of infectious disease physicians, hospitalists, intensivists, health services researchers, and data scientists. The Nosocomial Surveillance Task Force established 3 aims: (1) to monitor adherence to institutional infection control policies for PPE use and physical distancing intended to safeguard HCWs and optimize patient safety; (2) to monitor occupational exposure to and acquisition of COVID-19 by HCWs; and (3) to monitor patient safety related to COVID-19 by characterizing risk and rate of healthcare-associated COVID-19. Herein, we focus on the third aim of monitoring patient safety related to COVID-19; the other 2 aims are ongoing and results will be reported in detail elsewhere.

### Study setting

This investigation took place at a single, large, tertiary-care facility in Rochester, Minnesota, with a total of 2,081 beds on 2 neighboring hospital campuses.^9^ Over the period of analysis, elective surgical procedures were allowed by the state government and were performed at this institution. The institution had the following COVID-19 infection control policies in place: conversion of all patient rooms to single rooms, universal masking for staff and visitors, additional respiratory protection (N-95s or powered air-purifying respirators) for staff treating patients undergoing procedures classified as high risk for aerosol generation, masking of patients whenever they left their rooms, universal use of eye protection by staff when interacting with patients, limit of 1 visitor per hospitalized patient, and social distancing in common areas including waiting rooms, lobbies and elevators. During this period, the hospital census of COVID-19 patients ranged from 15 to 30, with a few new cases each day, and overall hospital occupancy ranged from 62% to 77% per week during the study. Polymerase chain reaction (PCR) testing for SARS-CoV-2 was widely available, and weekly test positivity rates in the community over the period of analysis ranged from 0.5% to 2.7%.

### Patients

**Fig. 1.**
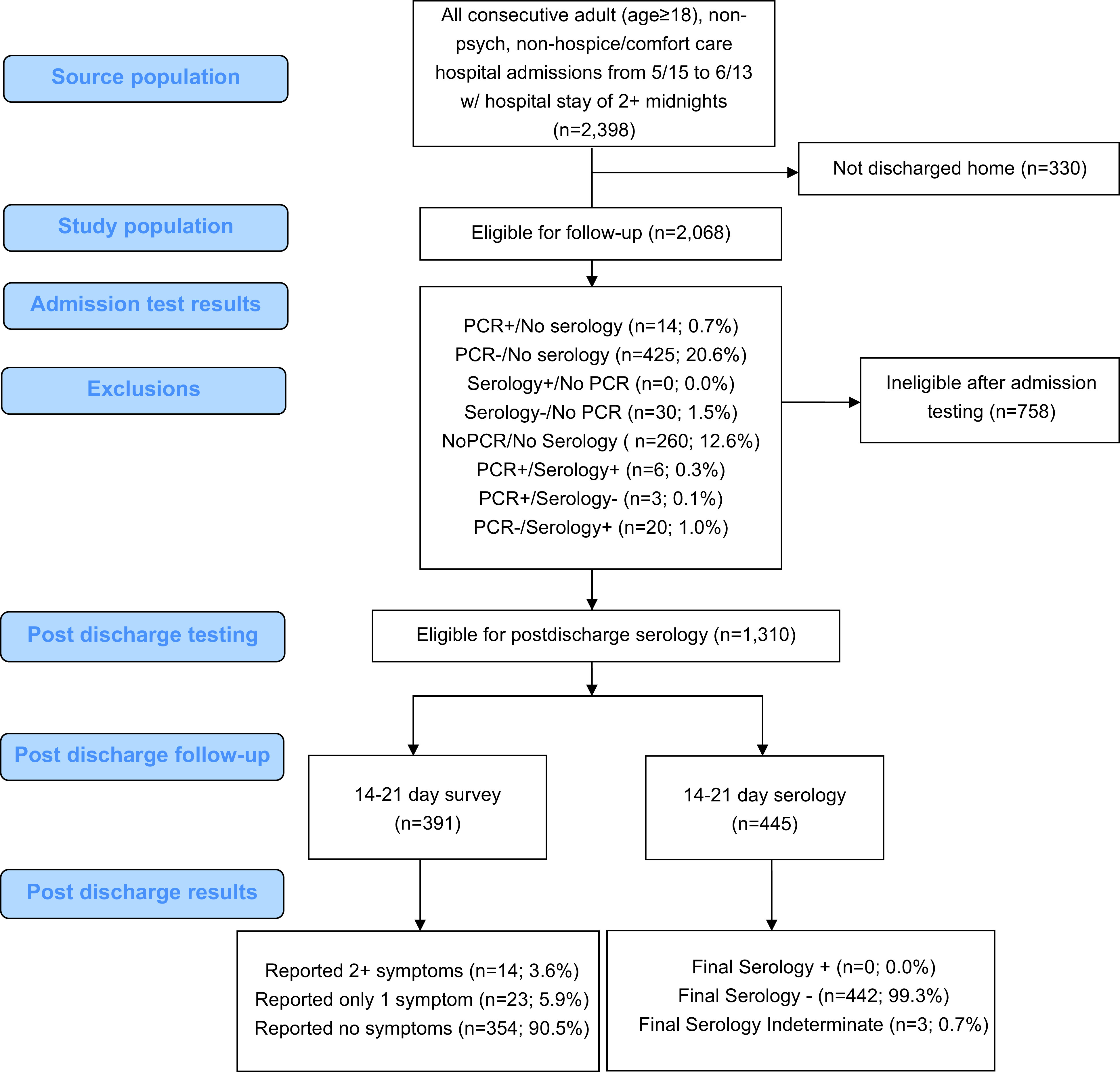
Nosocomial surveillance case-series flow diagram.

Patients were eligible for inclusion if they were hospitalized for 2 or more nights, were admitted on or after May 15, and were discharged by June 15 (Fig. 1). Therefore, the last eligible patients were admitted on June 13, stayed only 2 nights, and were discharged June 15. Patients admitted after June 13 and those who stayed in hospital past June 15 were not eligible. Patients hospitalized for 1 night or less were ineligible because our definition of hospital-acquired infection was based upon a minimum 2-night stay to best infer source (hospital vs community) of SARS-CoV-2.

### COVID-19 testing processes

#### Admission testing

Admission testing for by both PCR and serology was to be completed between 3 days prior to admission and 2 days following admission, and all patients were screened for symptoms suggestive of COVID-19 at or before admission to the hospital. Patients were able to refuse SARS-CoV-2 PCR testing, and it was not indicated in certain patient groups, like those who underwent recent sinonasal or skull base surgery, or those with severe thrombocytopenia. Symptomatic patients were subsequently managed based on the PCR test results, imaging results, and clinical syndrome. PCR testing results were available within 4–6 hours, and patients remained on isolation precautions until the PCR test was negative. Testing was performed using one of several SARS-CoV-2 PCR assays including a laboratory-developed test 8, ARIES SARS-CoV-2 Assay (Luminex Systems, Austin, TX) and the Roche cobas SARS-CoV-2 assay (Roche Molecular, Basel, Switzerland). All assays have received emergency use authorization (EUA) from the US Food and Drug Administration.

Admission SARS-CoV-2 IgG serology testing for the presence of antibodies was performed either before or within 48 hours of admission. Serology testing was performed on adult patients using the EUROIMMUN Anti-SARS-CoV-2 ELISA (IgG) (EUROIMMUN US, Mountain Lakes, NJ) and was confirmed with either the VITROS Anti-SARS-CoV-2 IgG test (Ortho-Clinical Diagnostics, Rochester, NY) or the Elecsys Anti-SARS-CoV-2 test (Roche Diagnostics, Mannheim, Germany). These assays have received emergency use authorization (EUA) from the US Food and Drug Administration. Pediatric patients, behavioral health patients, hospice patients, and comfort care patients were excluded from serologic testing.

**Fig. 2.**
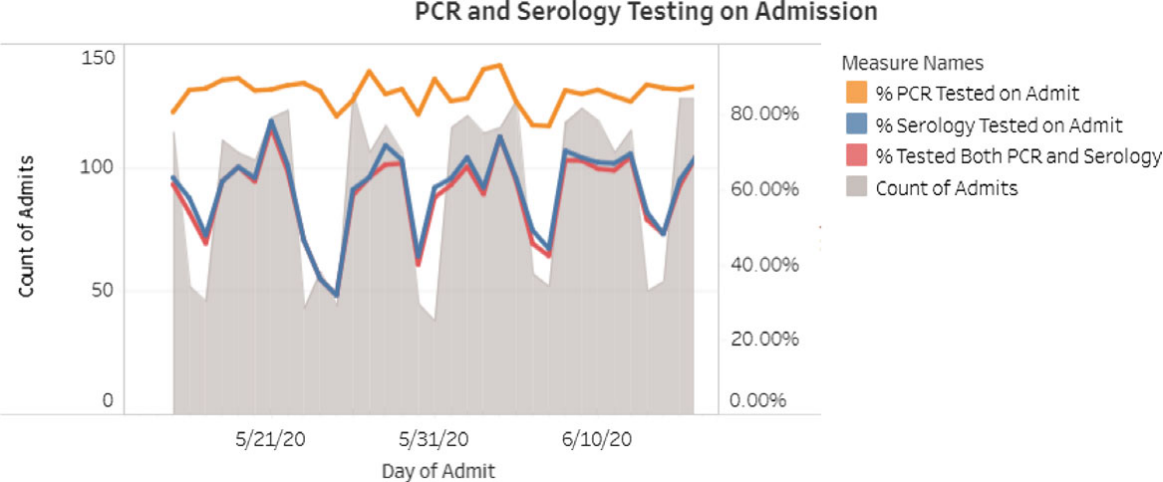
Admission PCR and serology testing rates as displayed on nosocomial surveillance dashboard.

The admission PCR and serology tests were ordered by nursing staff using a protocol. Patients who tested positive were managed by the patient’s primary service in consultation with the COVID-19 treatment team. Patients who developed new fever or respiratory symptoms while in the hospital were tested by a repeat SARS-CoV-2 PCR at the discretion of the healthcare team. Admission testing rates were updated and reviewed daily through an internally facing dashboard (Fig. 2).

#### Postdischarge evaluation

All patients with negative admission PCR and serology results were contacted for postdischarge serology testing between days 14 and 21 after hospital discharge. All eligible patients were sent a message regarding postdischarge serology testing through the patient portal at day 14 after discharge, with a reminder sent 3 days later. Patients who did not use the portal were contacted by phone. Notably, other serology testing initiated by patients who did or did not meet postdischarge surveillance eligibility criteria were also reviewed when observed in our institutional data.

Patients who elected to participate in the postdischarge evaluation either returned to one of our facilities for a venous blood draw or were provided with a mail out kit for blood draw to be performed at a location of their choosing, with the blood sample to be returned by mail to Mayo Clinic Laboratory for testing. Postdischarge serology tests that were positive via the EUROIMMUN Serology test were further tested via either the VITROS Anti-SARS-CoV-2 IgG test (Ortho-Clinical Diagnostics, Rochester, NY) or Elecsys Anti-SARS-CoV-2 test (Roche Diagnostics, Mannheim, Germany) using an orthogonal testing approach outlined on May 23, 2020, by the Centers for Disease Control and Prevention (CDC).^10^

Patients were also asked to complete a posthospitalization patient assessment questionnaire to assess whether they had had symptoms of COVID-19 or a positive test for COVID-19 within 2 weeks of hospital discharge (Appendix A online).

### Outcomes

In evaluation of risk for hospital-acquired COVID-19, prespecified criteria were used to identify definite healthcare-associated COVID-19, possible healthcare-associated COVID-19, and community-acquired COVID-19 (Table 1).

**Table 1.** Classification of COVID-19

Classification	Test Results
Community-acquired COVID-19	Positive PCR or serology at admission (day −3 to day 3 of hospital stay)
Definite healthcare-associated COVID-19	Negative PCR and serology testing at admission AND 1 of the following: Positive PCR test on ≥ day 7 of hospital stay OR Positive PCR test within 7 days after discharge from a hospitalization of at least 7 days duration OR Positive serology test on days 14–21 after hospital discharge
Possible healthcare-associated COVID-19	1. Negative PCR and serology testing at admission AND 1 of the following: Positive PCR test on days 4–6 of hospital stay OR Positive PCR test within 7 days after hospital discharge if the hospital stay was 3–6 days in duration 2. Missing PCR and serology at admission AND 1 of the following: Positive PCR test on of after day 7 of hospital stay OR Positive PCR test within 7 days after discharge from a hospital stay of at least 7 days duration 3. Missing or negative PCR and negative serology on admission AND Positive serology at days 14–21 after hospital discharge OR Positive PCR test on or after day 7 of hospital stay OR Positive PCR test within 7 days after discharge from a hospital stay of at least 7 days duration

### Analyses

Admission screening results for PCR and serology positivity were summarized and plotted over time (Fig. 2). Descriptive statistics, including number and percentage, were used to describe rates of community-acquired COVID-19, definite healthcare-associated COVID-19, possible healthcare-associated COVID-19, and posthospital questionnaire results. The 95% confidence intervals were calculated using the Wilson 95% CI method without continuity correction^11^ and were used to estimate rates of infection to account for small sample sizes.

For patients eligible for postdischarge serology testing (negative on PCR and serology testing at admission and discharged to home), demographics and hospitalization variables were compared with those patients who did and did not complete postdischarge serology testing (Table 2).

**Table 2.** Characteristics of All Patients Eligible for Postdischarge Serology

Characteristic	Completed Postdischarge Serology (n=445; 34.0%), No. (%)	Did Not Receive postdischarge Serology (n=865; 66.0%), No. (%)	*P* Value^a^
**Age, y**			.02
18–49	107 (24.0)	269 (31.1)	
50–64	144 (32.4)	232 (26.8)	
65–74	131 (29.4)	216 (25.0)	
75–84	52 (11.7)	121 (14.0)	
85+	11 (2.5)	27 (3.1)	
Median (IQR)	63 (50–70)	62 (43–71)	.24
**Race/Ethnicity**			.16
White, non-Hispanic	417 (93.7)	776 (89.7)	
Black	8 (1.8)	27 (3.1)	
Asian	8 (1.8)	19 (2.2)	
Hispanic of any race	2 (0.5)	11 (1.3)	
Other/Unknown	10 (2.3)	32 (3.7)	
**Sex**			.43
Male	235 (52.8)	437 (50.5)	
Female	210 (47.2)	428 (49.5)	
Length of stay, median d (IQR)	3 (2–5)	3 (2–5)	.63
ICU utilization, yes or no	92 (20.7)	218 (25.2)	.07
**Final postdischarge serology**			
Positive	0 (0.0)	N/A	
Negative	442 (99.3)		
Indeterminate^b^	3 (0.7)		
Days from discharge to follow-up serology, median (IQR)	15 (14–17)	N/A	
**Postdischarge survey**			.38
2+ symptoms	7 (1.6)	7 (0.8)	
1 symptom only	13 (2.9)	10 (1.2)	
0 symptoms	146 (32.8)	208 (24.0)	
Missing	279 (62.7)	640 (74.0)	

a χ^2^ test for categorical variables, Wilcoxon rank-sum test for continuous variables.

b No subsequent tests available.

In determining the cohort size necessary to evaluate hospital-acquired infection, we estimated that a sample size of 274 patients would be sufficient to detect a 2.0% infection rate with 80% power and α = 0.05. Incidence of hospital-acquired COVID-19 was calculated along with a 95% confidence interval.

This initiative was reviewed by the Mayo Clinic Institutional Review Board and was considered a quality improvement project.

## Results

### Admission testing

PCR testing for COVID-19 upon admission was completed for 3,043 of 3,453 admitted patients (85.6%) during the study period. Among them, 56 patients (1.8%; 95% CI, 1.4%–2.4%) tested positive; and positivity rates remained relatively constant over time. Notably, these 56 patients do not represent all COVID-19–positive admissions over the study period because not all known COVID-19 admissions met the study inclusion criteria.

#### Community-acquired COVID-19

In total, 9 asymptomatic patients were identified through admission PCR testing (0.3% of admissions). An additional 47 cases were identified in patients symptomatic of COVID-19 (1.5% of admissions).

### Postdischarge evaluation

#### Eligibility and completion

Protocol-eligible patients included adults hospitalized for 2 or more nights and discharged to their homes (n = 2,068); patients were roughly split between surgical (51.3%) and nonsurgical patients. Of the 2,068 protocol-eligible patients, 1,778 (86.0%) completed admission PCR testing with or without serology testing, and 1,339 (64.7%) completed both PCR and serology testing at admission.

Patients negative by PCR and serology (1,310, 97.8%) were contacted for postdischarge serology and symptom assessment. Half (48.7%) of the patients in our postdischarge evaluation cohort were female, and the median age was 62 years (interquartile range, 46–71).

Postdischarge serology testing was completed by 445 (34.0%), and 391 (29.8%) completed the posthospitalization patient assessment questionnaire. Upon comparison of demographic and clinical factors for eligible patients who did and did not complete postdischarge serology testing, patients completing postdischarge serology testing were of similar median age, race and sex and experienced similar lengths of stay (median, 3 days in both groups) and ICU utilization (Table 2).

### Outcomes

#### Definite healthcare-associated COVID-19

Of the 1,310 patients who tested negative by PCR and serology at admission, no patients met the a priori definition of hospital-associated COVID-19. No patient tested positive by PCR on or after day 7 of hospital admission when including all admitted eligible patients (95% confidence interval: 0.0%–0.3%) or when limited to the 201 patients with a hospital stay of ≥7 days (95% confidence interval [CI]: 0.0%–1.9%).

Of the 445 patients who tested negative by PCR and serology at admission and completed postdischarge serology, no patients seroconverted (0.0%; 95% CI, 0.0%–0.9%). Two patients initially met the a priori definition of hospital-associated COVID-19 (0.4%; 95% CI, 0.1%–1.6%) based on postdischarge serology testing using the EUROIMMUN assay. The positive serum samples were retested using the orthogonal testing approach outlined by the CDC^10^ with the VITROS assay (n = 1) and the Elecsys assay (n = 1) and were both negative. Neither of these patients had any symptoms suggestive of COVID-19.

#### Possible healthcare-associated COVID-19

No patients were ultimately determined to have possible hospital-associated COVID-19. However, 5 patients were flagged as meeting the a priori criteria for possible hospital-associated COVID-19 because they were missing PCR tests during the admission testing period of day −3 to day 2 of hospital admission, yet subsequently, they tested positive with PCR and/or serology. Upon further review, all patients had known positive PCR test results for COVID-19 4 or more days prior to admission. Given their established prior COVID-19, these patients were no longer considered to have possible nosocomial infections. However, this information did verify that the electronic rules worked well to identify patients with a potential hospital-associated COVID-19 who merited additional review. No patients who were negative on admission later had positive PCR tests on days 4–6 of hospital stay or had a positive PCR test within 7 days of hospital discharge following a 3- to 6-day hospitalization.

#### Posthospitalization patient assessment questionnaire results

Of the 391 patients who completed the postdischarge questionnaire 14–21 days after discharge, 37 (9.5%) reported at least 1 of the following symptoms: headache (2.0%), diarrhea (1.0%), fever (1.3%), muscle aches (1.8%), nausea (0.8%), cough (0.3%), loss of taste (0.5%), shortness of breath (0.0%), vomiting (0.3%), sore throat (0.3%), fast breathing (0.0%). Moreover, 14 patients (3.6%) reported 2 or more symptoms, and 4 patients (1.0%) reported 3 or more symptoms. An infectious disease physician reviewed records for each patient who reported postdischarge symptoms and found alternative explanations for symptoms, symptoms not compatible with COVID-19, or negative testing for COVID-19.

## Discussion

In this evaluation at a large, Midwestern, tertiary-care facility, with ongoing, albeit low, rates of community transmission, we have demonstrated that complex medical care in the hospital can be delivered safely in the setting of multiple institutional infection control policies. Our framework for assessing the risk of hospital-associated COVID-19 will inform future investigations at our institution, and our approach and definitions may additionally be utilized by other institutions. We identified zero nosocomial infections, similar to results from another study of nosocomial COVID-19 in an overlapping time period, which also reported low (0.1%) risk of infection.^12^ The very low risk of healthcare-associated COVID-19 should reassure patients that it is safe to seek necessary medical care including hospitalization during the COVID-19 pandemic.

In preparation for gradual resumption of elective care while COVID-19 was still circulating, we established new infection control requirements for staff, patients, and visitors. We created tools to measure adherence to these requirements, including dashboards to display these results, and we provided immediate feedback about deficiencies through clinical communication channels. Simultaneously, we measured the rate of healthcare-associated COVID-19 to assess whether these strategies were effective.

Hospitals and academic medical centers must determine how to safely operate in the COVID-19 environment,^13^ not only for patient health but also to remain financially solvent^1,2^ and to protect employees when providing care.^14–17^ Healthcare-associated infections may be reduced when infection control measures such as testing of employees and patients, PPE use, increased cleaning and disinfection efforts, isolation of infected patients, physical distancing in lobbies and other public areas, and contact tracing are implemented,^8,18,19^ and healthcare-associated infections can be avoided when telehealth is used.^20^

At our institution, we have implemented structures and processes to reduce the risk of healthcare-associated COVID-19 to our patients and employees. We have aimed to move all patients to single rooms, minimizing the risk of cohousing non–COVID-19 patients with a patient with undiagnosed COVID-19. Visitors have been limited to 1 specific individual per patient for the patient’s entire hospital stay. Universal masking is required of all visitors and employees, and all are screened daily for severe acute respiratory coronavirus virus 2 (SARS-CoV-2) exposure, COVID-19 symptoms, and temperature. Employees with symptoms have access to testing, and employees with known exposures are quarantined with pay. These measures may underlie the low observed rate of nosocomial infection in our hospitalized patients.

Based upon these results and an otherwise developing institutional knowledge base, we have discontinued routine serology testing both at admission and after dismissal. We continue to screen patients at admission and to ask patients to call us if they develop symptoms of COVID-19 after discharge. We have set up a mechanism to alert the infection control team about potential healthcare-associated SARS-CoV-2 patient infections based on the definitions outlined in Table 2 (excluding serology testing criteria). The alerts prompt additional review by infection control staff.

Studies of nosocomial transmission of SARS-CoV-2 from other geographic areas are just beginning to be published. In a Belgian hospital, 1 COVID-19 patient is suspected to have infected 4 other hospitalized patients, with a total of 31 probable nosocomial infections in hospitalized patients during the first weeks of the pandemic.^7^ A London hospital suspected that 15% of its hospitalized COVID-19 patients from early March through mid-April contracted the disease from the healthcare environment (11% definite and 4% probable).^8^ Other studies have evaluated the risk of nosocomial infection within HCWs.^16,18,19,21^ However, none of these publications evaluated the risk of healthcare-associated COVID-19 in a cohort of patients hospitalized for non–COVID-19 indications.

As mentioned above, a recently published study did evaluate the incidence of COVID-19, at Brigham and Women’s Hospital in Boston, Massachusetts, and also found low risk of hospital infection.^12^ Subsequent to their period of analysis and article publication, a press release^22^ was issued regarding an outbreak at this facility, underscoring the importance of continued vigilance. As of October 16, 2020, the outbreak involved 15 patients and 42 employees related to 2 hospital units. They attributed the outbreak to several factors including a highly infectious source patient, very high viral loads in several other patients, and provider inconsistencies with eye protection during patient care and masking during breaks.

Through our ongoing electronic alert system, we identified 3 healthcare-associated COVID-19 cases between June 15 and September 30. Infection control investigations identified the likely sources of infection as a HCW who tested positive and had cared for the patient, a visitor who visited the patient while infectious, and an unknown source despite extensive testing of the patient’s visitor and all HCWs who cared for the patient. We continue to monitor adherence with masking and eye protection use and so far have not identified patient-to-HCW transmission. Electronic surveillance is ongoing and quite useful as community spread of SARS-CoV-2 continues, with potential risk of corresponding hospital transmission.

Although the risk of nosocomial infection when hospitalized in our institution is low, there exists a separate yet demonstrable risk of delaying necessary medical and preventive care, and a number of studies have observed decreased use of health services. A poll conducted by the Kaiser Family Foundation in mid-May 2020 reported that nearly half (48%) of Americans polled said that they or a family member skipped or delayed medical care due to the COVID-19 environment. As a result, 11% of respondents reported that health worsened for the person who delayed medical care.^4^ Reports of cancer patients having or choosing to delay care during COVID-19 are devastating^23^ and could lead to thousands of additional cancer deaths as a result.^24^ Furthermore, a study of 24 emergency departments (EDs) across 5 states found rapid declines in the number of ED visits beginning the week of March 11, 2020, and those who were willing to approach the ED were likely of higher acuity because they were more likely to be admitted than in historical data.^5^ Efforts to enhance the return of patients to emergency, preventive, and other necessary forms of health care are imperative.

This study has several limitations. This evaluation was a quality improvement initiative focused on patient safety at a single institution; therefore, our results may not be generalizable to other hospitals overall or those of varying bed size, patient or procedural mix, or geographic location. However, our results are in line with another institution’s report.^12^ Furthermore, during the study period, community spread was moderate in our surrounding area compared to other regions, although the existence of PCR testing upon admission may make community prevalence a lesser concern. Results warrant further study in areas with higher community rates of infection. As with nearly any laboratory test, both false-positive and false-negative results may occur. We used the orthogonal testing algorithm for postdischarge serology results (ie, confirmation of positive serology tests with a second assay), but we did not confirm positive admission serology results, and it is likely that some of the baseline serology results were false positives. However, baseline false-positive results did not impact the overall findings of this study, given that the purpose of the baseline serology was to observe seroconversion after discharge.

Our participation in postdischarge serology testing was 34.0%, which is low but may be understandable because patients recovering from hospitalizations may be unwilling to leave their homes during a pandemic to return to a healthcare facility for a laboratory draw that may not impact their individual care. Notably, differences between patients who did and did not participate in this postdischarge testing were minimal; however, to account for this smaller sample size, our conclusions are built upon confidence intervals rather than point estimates. In addition, since this analysis included patients hospitalized for at least 2 nights, its implications for outpatient care are unclear. However, it is the first step toward establishing confidence in accessing health care during the COVID-19 pandemic, and to our knowledge is the first study to incorporate postdischarge follow-up for evidence of COVID-19 rather than limiting observation to the hospitalization alone.

In conclusion, in this evaluation of risk of nosocomial infection with SARS-CoV-2 in hospitalized patients, low likelihood was established. Patients should be assured that accessing health care during this COVID-19 era for non–COVID-19 reasons is safe provided that certain safeguards, such as universal masking, physical distancing, and screening, are in place. Other institutions may assess their patient safety with the provided framework and definitions of definite and possible healthcare-associated COVID-19 as they move toward increasing elective surgeries and admissions.

## Appendix A. Mayo Clinic Posthospitalization Patient Assessment Questionnaire

A. Have you had any of the following within the past 14 days?
  - Fever ≥ 37.8°C (100°F)
  - New cough
  - New or worsening shortness of breath or difficulty breathing
  - Chills or repeated shaking with chills
  - Muscle pain
  - Headache
  - Sore throat
  - Loss of sense of taste or smell
  - Diarrhea
B. Within the past 14 days have you been exposed to someone who was diagnosed with COVID-19?
